# Supplementary material for: Sumoylation regulates FMRP-mediated dendritic spine elimination and maturation
Source: Nat Commun. 2018 Feb 22;9:757. doi: 10.1038/s41467-018-03222-y (PMC5823917; doi:10.1038/s41467-018-03222-y)
Supplement: Supplementary file 1 — Supplementary Information [file 41467_2018_3222_MOESM1_ESM.pdf]

Supplementary Information

Sumoylation regulates FMRP-mediated dendritic spine elimination and maturation

Khayachi et al.

Supplementary Information

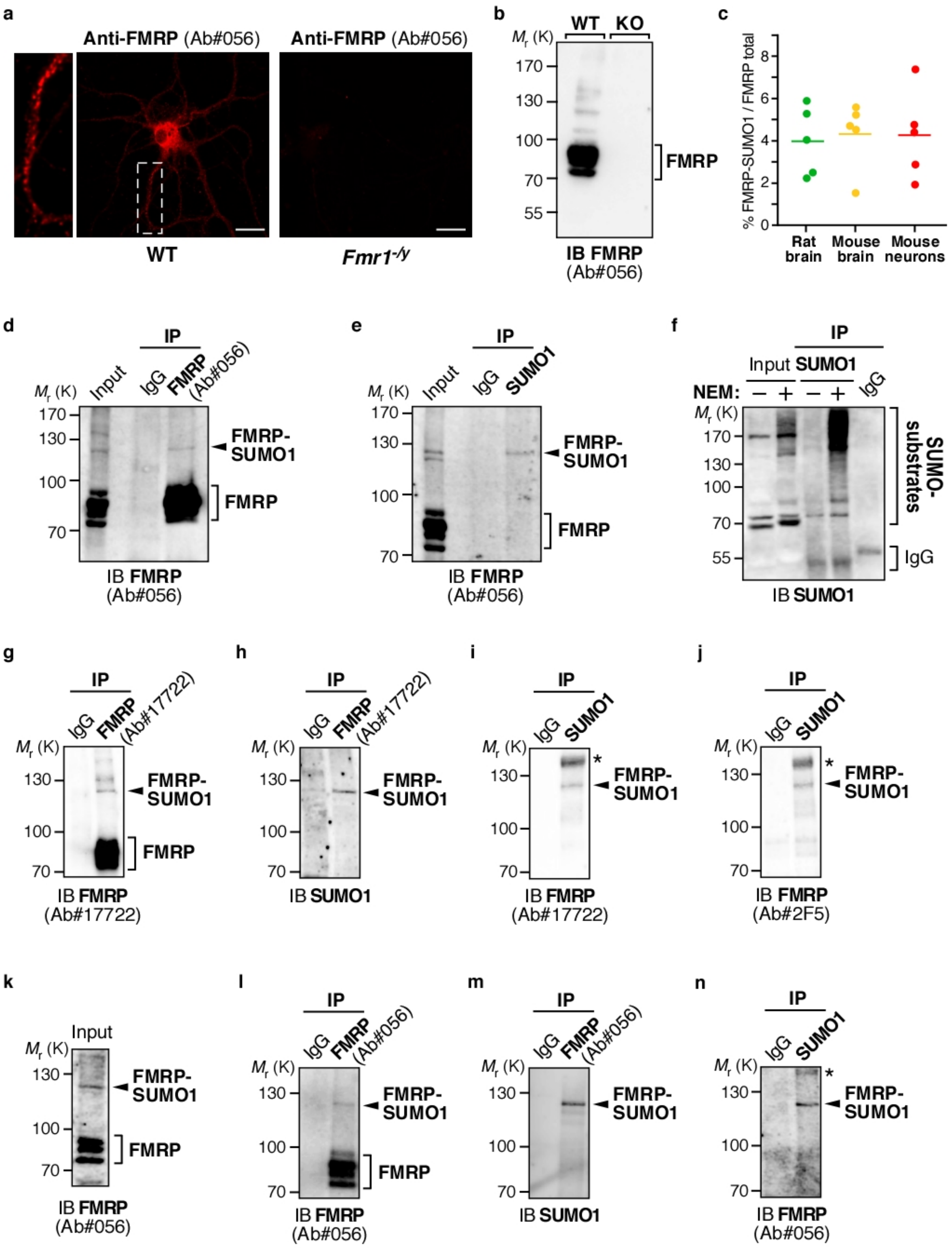

Supplementary Figure 1

Supplementary Figure 1. Characterization of the custom anti-FMRP (Ab#056) antibody and additional evidence for the *in vivo* and *in vitro* sumoylation of FMRP. (a) Immunostaining of

the endogenously expressed FMRP with the custom anti-FMRP antibody (Ab#056; 1 μg/mL) in WT mouse hippocampal neurons (18 DIV). Control immunostaining experiment with the custom anti-FMRP antibody (Ab#056; 1 μg/mL) on 18 DIV *Fmr1*$^{-/y}$ hippocampal neurons. Bar, 20 μm. **(b)** Representative immunoblot with the anti-FMRP (Ab#056) antibody of P7 post-nuclear WT and *Fmr1*$^{-/y}$ mouse brain extracts. **(c)** Scatter plots of the ratio between the intensity of FMRP-SUMO1 and the total amount of FMRP from input lanes (n=5 for each conditions) measured on FMRP immunoblots obtained from rat brain, mouse brain and mouse neuronal homogenates. Statistical analysis using an ANOVA with a Bonferonni post-test revealed no significant differences between the 3 conditions. **(d,e)** Immunoblots with the anti-FMRP (Ab#056) of NEM-treated P7 rat brain extracts subjected to immunoprecipitation with FMRP (Ab#056) **(d)** or anti-SUMO1 **(e)** antibodies. Input and control IgG lanes are also shown on the blots. **(f)** Control experiments demonstrating the protective role of NEM on protein sumoylation. Control and NEM-treated P7 rat brain homogenates were subjected to immunoprecipitation with anti-SUMO1 antibodies. Input and control IgG lanes are also shown on the blots. **(g,h)** Immunoblots with the anti-FMRP (Ab#17722; **g**) or anti-SUMO1 **(h)** antibodies of NEM-treated P7 rat brain extracts subjected to immunoprecipitation with FMRP (Ab#17722) antibody or control IgG. **(i,j)** Immunoblots with the anti-FMRP (**i**, Ab#17722; **j**, Ab#2F5-1) antibodies of NEM-treated P7 rat brain extracts subjected to immunoprecipitation with SUMO1 antibody or control IgG. *Non-specific band. **(k)** Input blot with the anti-FMRP (Ab#056) antibody on homogenates from 18 DIV NEM-treated rat cortical neurons used in **l** and **m**. **(l,m)** Immunoblots with the anti-FMRP (Ab#056; **l**) or anti-SUMO1 **(m)** antibodies on neuronal extracts shown in **(k)** subjected to immunoprecipitation with FMRP (Ab#056) antibody or control IgG. **(n)** Immunoblot with anti-FMRP (Ab#056) antibody on homogenates from 18 DIV NEM-treated cortical neurons and subjected to immunoprecipitation with SUMO1 specific antibody or control IgG. *Non-specific band.

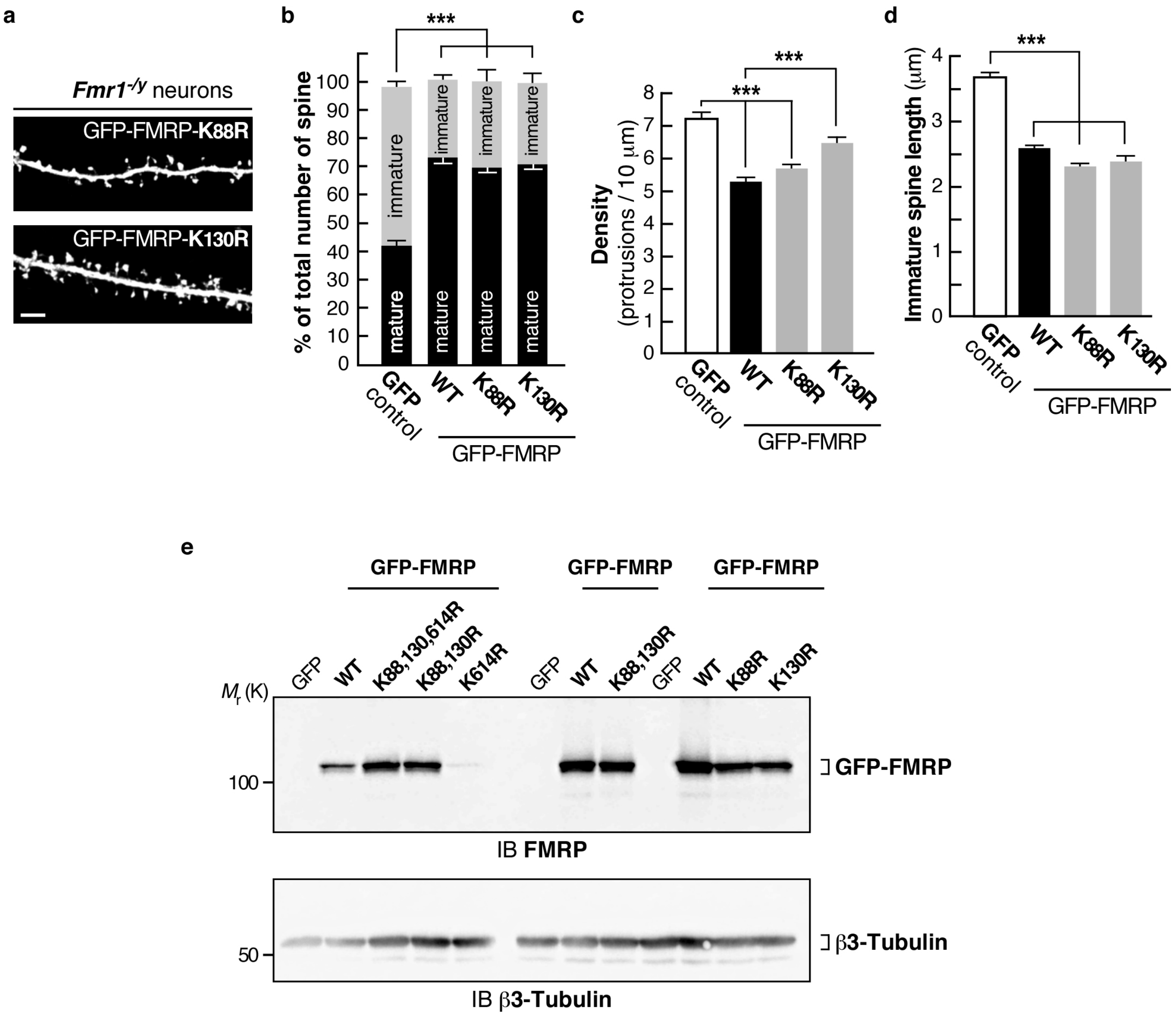

Supplementary Figure 2

Supplementary Figure 2. (a) Representative confocal images of dendrites from transduced *Fmr1*$^{-/y}$ neurons expressing the K88R or K130R mutant forms of GFP-FMRP for 24h. Bar, 5 µm. Histograms show the relative proportion of mature and immature spines **(b)** and the density of the protrusions **(c)** in the indicated conditions. **(d)** Histograms of immature spine length measured from *Fmr1*$^{-/y}$ neurons expressing the indicated constructs. Data shown in **b-d** are the mean ± s.e.m. Statistical significance in **b-d** was determined by a one-way analysis of variance (ANOVA) with a Bonferroni post-test. N = ~4500 spines per condition from 4 independent experiments. ***p<0.001. **(e)** Relative protein expression levels of the WT and mutant forms of GFP-FMRP in *Fmr1*$^{-/y}$ transduced neurons. Input lanes for β3-tubulin are also shown.

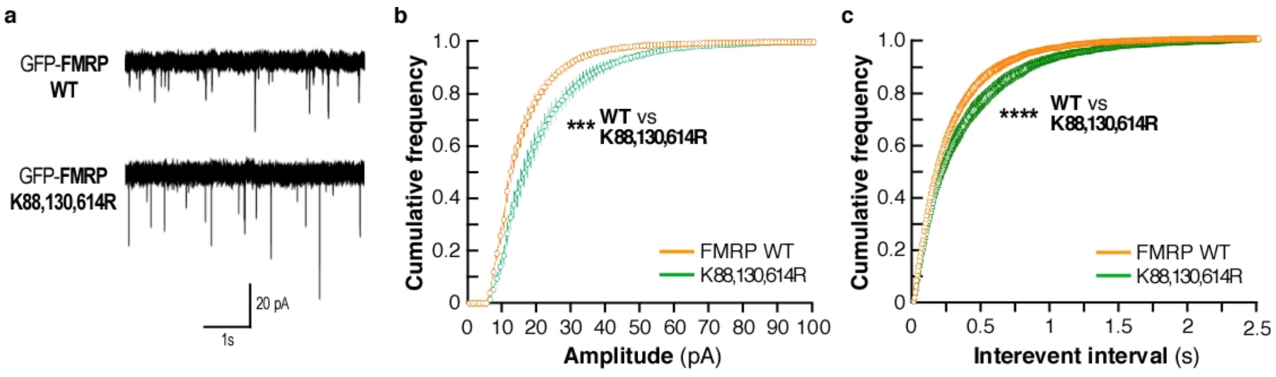

Supplementary Figure 3

Supplementary Figure 3. Sumoylation is involved in the regulation of FMRP function. **(a)** Representative sample traces from **WT** and **K88,130,614R** GFP-FMRP-positive *Fmr1*$^{-/y}$ neurons. Cumulative frequency for amplitudes **(b)** and interevent intervals **(c)** of mEPSCs recorded from GFP-FMRP **WT** and **K88,130,614R** expressing neurons (20 and 19 cells respectively from 4 different cultures). Statistical significance was determined by analysis of the amplitude and interevent interval distributions using a Kolmogorov-Smirnov test. ***p<0.001; ****p<0.0001.

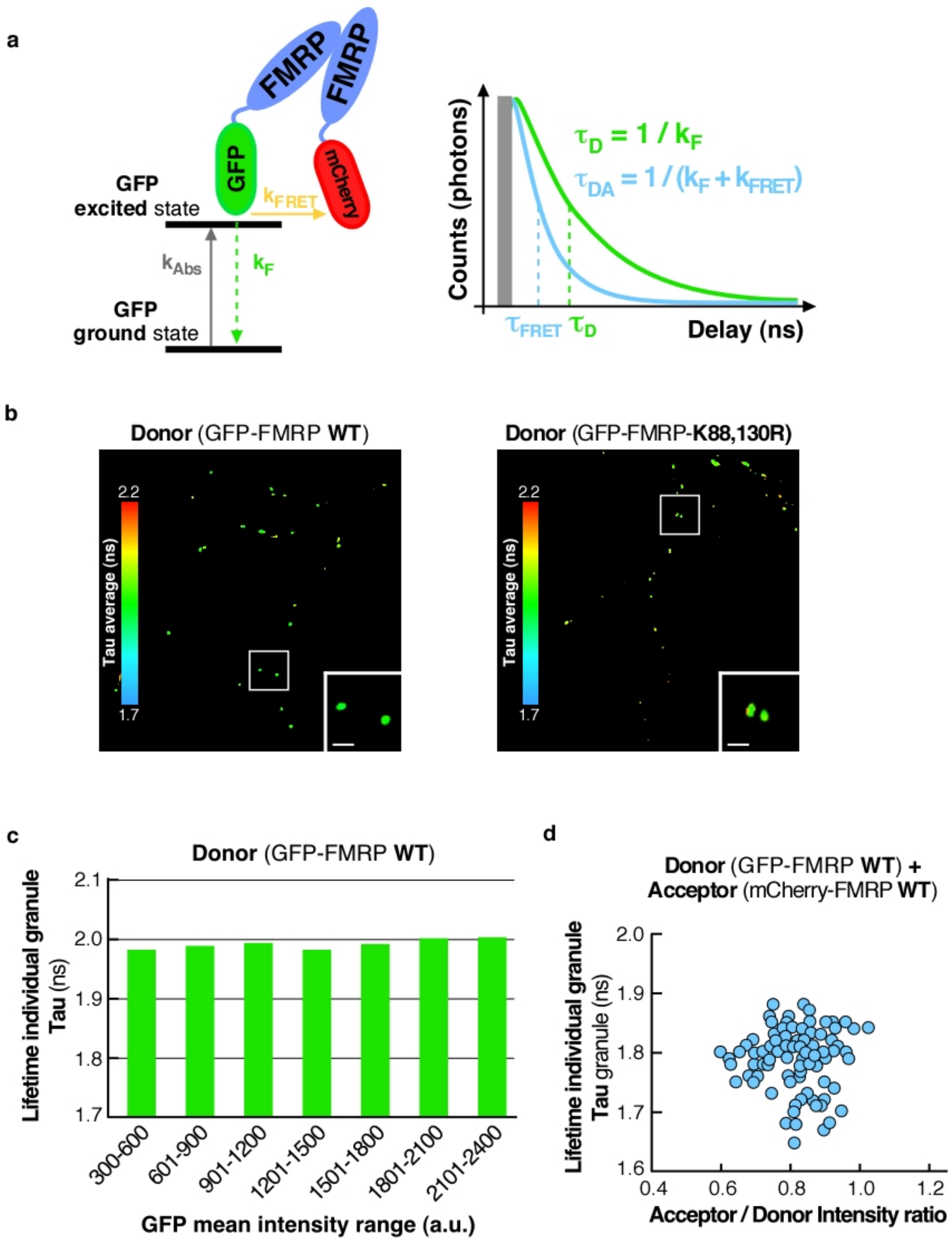

Supplementary Figure 4

Supplementary Figure 4. Control data for the FLIM experiments. (a) Original schematic representation of FLIM. When photophysical criteria are met (spectral overlap, distance…) Donor GFP in the excited state can transfer its energy to the acceptor mCherry. The possibility to relax by FRET provides an additional pathway to the radiative relaxation (emission of a photon).

Therefore the time spent by GFP in the excited state (fluorescence lifetime) is shortened in presence of FRET *i.e.* when the two proteins interact. Fluorescence lifetime can be expressed as a function of the kinetic constants of the relaxation processes (k_F and k_{FRET} for fluorescence and FRET respectively). As a result, the intensity decay in the presence of Acceptor (blue curve) is faster than in the case of Donor alone (green curve). **(b)** FLIM images of GFP-FMRP (Donor, D) alone in the WT and K88,130R forms. Fluorescence lifetime is represented using a pseudo-color scale ranging from 1.7 to 2.2 ns. Insets show representative clusters for each condition. **(c)** Average lifetime of individual granules is invariant with regards to the granule GFP intensity. The histogram represents the average lifetime of GFP-FMRP WT individual granules (n=172) over 3 independent experiments. Fluorescence lifetime was calculated for granules with sufficient photon counts (at least 500 Cnts/pixel and 10^4 Cnts/granule) and which were acquired with a count rate inferior to 4 MHz as detailed in the Methods. **(d)** Average lifetime of individual granules is randomly distributed over A/D intensity ratio. Represented granules correspond to neurons expressing WT forms of GFP-FMRP and mCherry-FMRP (n=171 granules over 2 independent experiments).

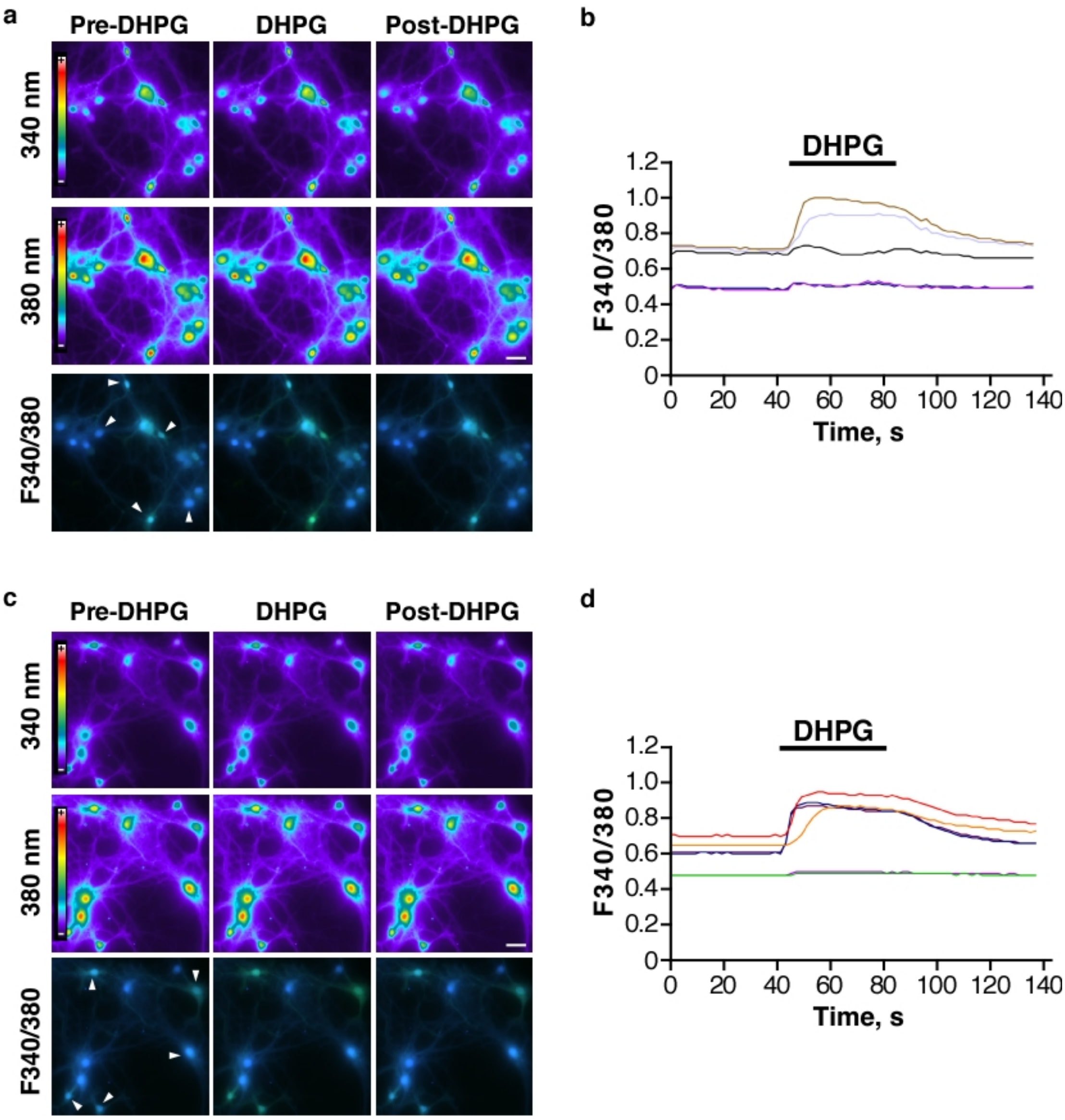

Supplementary figure 5

Supplementary Figure 5. Control intracellular calcium responses evoked by an mGlu5R activation in mouse WT and *Fmr1$^{-/y}$* neurons. (a,c) Representative pseudocolored images of Fura-2 fluorescence ratio (340/380 nm) showing WT **(a)** and *Fmr1$^{-/y}$* neurons **(c)** before (pre-DHPG), during (DHPG) and after (post-DHPG) an mGlu5R agonist stimulation. Bar, 20 µm. **(b,d)** Representative traces of Fura-2 fluorescence 340/380 nm ratio (F340/380) overtime showing an increase in intracellular Ca^{2+} concentration both in WT **(b)** and *Fmr1$^{-/y}$* **(d)** neurons exposed to DHPG. Arrowheads in **(a)** and **(c)** point to neurons in which the F340/380 ratio is shown in **(b)** and **(d)** respectively.

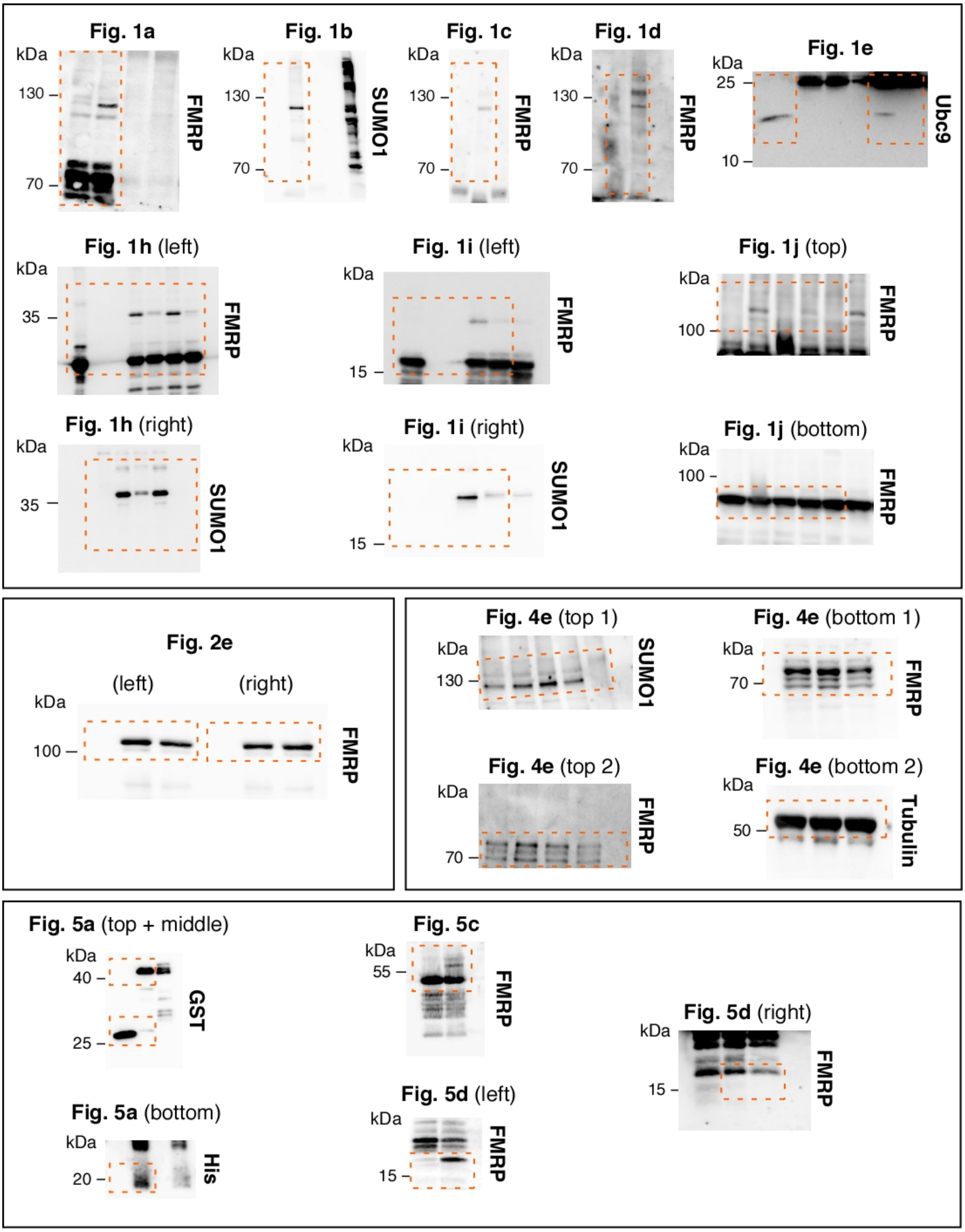

Supplementary figure 6

Supplementary Figure 6. Original uncropped blots. Orange boxed regions represent the portion used in **figures 1 to 5**.

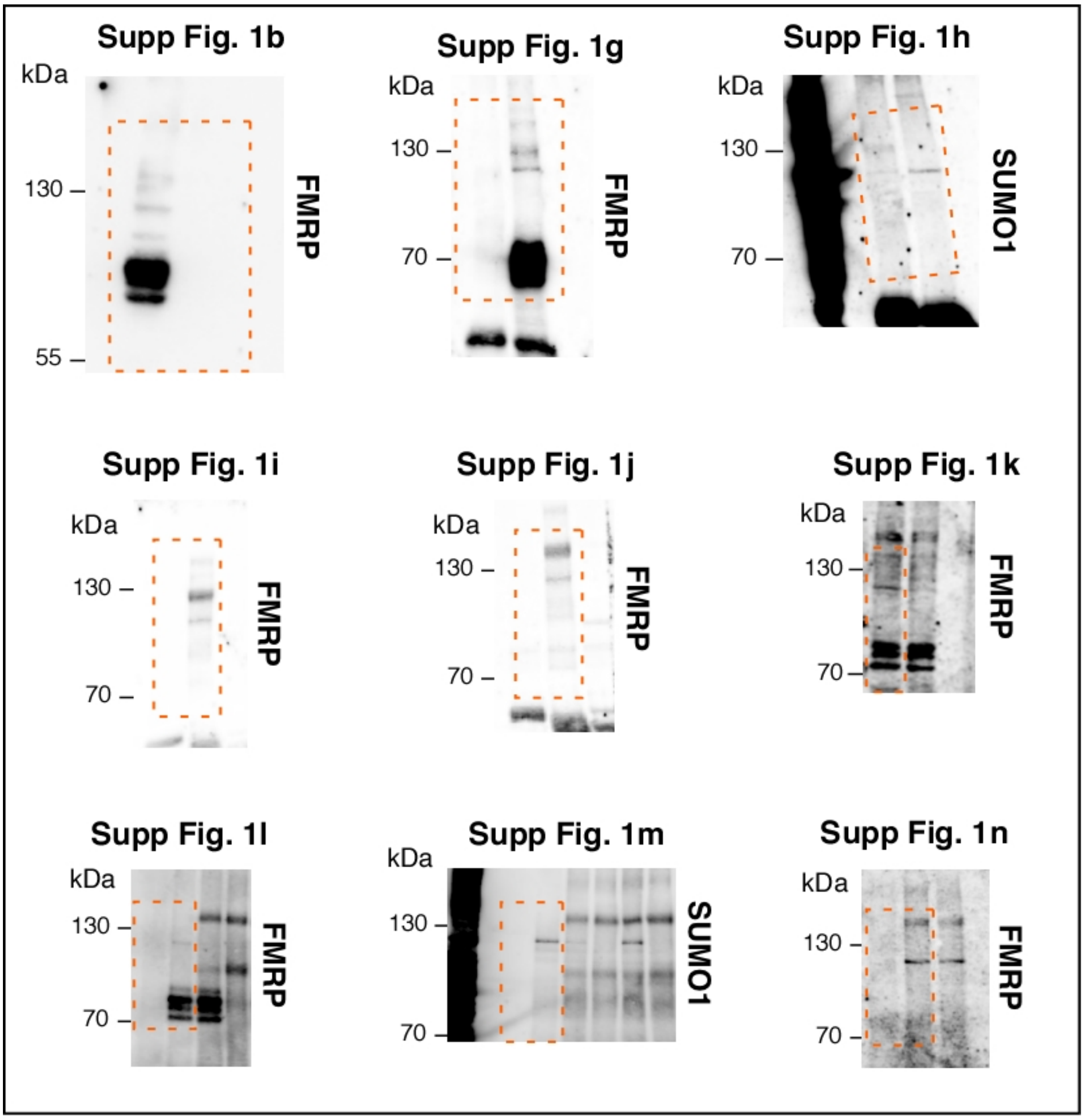

Supplementary figure 7

Supplementary Figure 7. Original uncropped blots. Orange boxed regions represent the portion used in the **Supplementary figure 1**.
